# The Development of a Mobile Application to Support Peripheral Health Workers to Diagnose and Treat People with Skin Diseases in Resource-Poor Settings

**DOI:** 10.3390/tropicalmed3030102

**Published:** 2018-09-15

**Authors:** Liesbeth F. Mieras, Anna T. Taal, Erik B. Post, Alcino G. Z. Ndeve, Colette L. M. van Hees

**Affiliations:** 1 Technical Department, Netherlands Leprosy Relief, 1090 HA Amsterdam, The Netherlands; A.Taal@Leprastichting.nl; 2 Challenge TB, KNCV Tuberculosis Foundation, Jakarta 12870, Indonesia; Erik.Post@KNCVtbc.org; 3 Technical Department, Netherlands Leprosy Relief, Maputo, Mozambique; AlcinoNdeve@nlrmoz.org; 4 Department of Dermatology, Erasmus Medical Centre, 3000 CA Rotterdam, The Netherlands; C.vanHees@ErasmusMC.nl

**Keywords:** skin diseases, mobile phone application, NTDs, dermatology, mHealth, leprosy

## Abstract

The high prevalence of skin diseases in resource-poor settings, where health workers with sufficient knowledge of skin diseases are scarce, calls for innovative measures. Timely diagnosis and treatment of skin diseases, especially neglected tropical diseases (NTDs) that manifest with skin lesions, such as leprosy, is crucial to prevent disabilities as well as psychological and socioeconomic problems. Innovative technological methods like telemedicine and mobile health (mHealth) can help to bridge the gap between the burden of skin diseases and the lack of capable staff in resource-poor settings by bringing essential health services from central level closer to peripheral levels. Netherlands Leprosy Relief (NLR) has developed a mobile phone application called the ‘SkinApp’, which aims to support peripheral health workers to recognize the early signs and symptoms of skin diseases, including skin NTDs, and to start treatment promptly or refer for more advanced diagnostic testing or disease management when needed. Further research is needed to determine how greatly mHealth in general and the SkinApp in particular can contribute to improved health outcomes, efficiency, and cost-effectiveness.

## 1. Introduction

### 1.1. Short Communication

This article is written as a short communication, describing the ongoing development process of a mobile phone application that supports peripheral health workers in diagnosing and treating skin diseases in resource-poor settings.

### 1.2. Epidemiology of Skin Diseases in Low- and Middle-Income Countries

It is known that skin diseases are highly prevalent, particularly in resource-poor areas, where children are disproportionally affected—though there is a lack of systematically-collected data supporting this [1,2]. The epidemiology of skin diseases varies per country and within countries, but available data show that in rural areas, usually more than 50% and sometimes as much as 80% of the population has a skin condition; especially in areas endemic for conditions like scabies, onchocerciasis or tinea capitis [1]. The Global Burden of Disease (GBD) Study 2013 indicated that skin conditions were the fourth leading cause of disability worldwide [3].

Neglected topical diseases (NTDs) that manifest in skin lesions like leprosy and lymphatic filariasis (see Table 1) are among the skin diseases that often cause disabilities such as permanent wounds, contractures, and advanced lymphedema. Timely diagnosis and treatment can prevent permanent disabilities, but skin NTDs are usually not the most prevalent skin diseases, and are therefore more likely to be missed by health workers with limited or no dermatology training.

Skin conditions are usually not fatal, but it is important to recognize those that may be, such as blistering diseases. They can also be a sign of an underlying medical condition that might be life-threatening, such as HIV/AIDS. In view of the HIV/AIDS epidemic, the importance of dermatology cannot be underestimated, as 90% of HIV/AIDS patients develop a skin problem (see Table 1) during the course of their illness, and often initially present with a skin condition [4].

### 1.3. Psycological and Socioeconomic Burden

Physical problems caused by skin conditions can lead to deterioration of the quality of life. Additionally, skin diseases often cause psychological problems such as anxiety, depression, and exclusion. A study in Colombia to determine the impact of skin diseases on the quality of life found that even the most localized or asymptomatic skin lesion leads to a disruption at some level of patient wellness [5]. The high visibility of skin diseases contributes to the likelihood of stigmatization, and may even result in unemployment [6]. Skin diseases can negatively impact productivity at school and work, while a considerable amount of household money has to be spent on treatment that is often ineffective due to insufficient availability of health workers with basic dermatology training at the peripheral level [7]. The economic impact of accessing care and rehabilitation services can be substantial, especially when the condition is chronic or leads to disabilities [8].

### 1.4. Capacity Gap

Studies have shown that a minimum of 10% of the consultations at peripheral healthcare level relates to common skin diseases [9,10]. Unfortunately, healthcare workers often lack the knowledge and skill to diagnose and treat skin diseases due to lack of training [7,9]. There is a need to bridge the gap between the prevalence of skin conditions at community level and the availability of capable staff in peripheral health facilities. An increased capacity to diagnose and treat skin diseases will contribute to the timely diagnosis and treatment of skin diseases in general, and skin NTDs in particular, and thus to the prevention of chronic conditions and disabilities.It will also contribute to timely and appropriate referral. Seth et al. call for increased efforts to address this problem, especially in low-resource settings, using recent innovations to help provide dermatological care to underserved regions in a cost-effective manner [11].

## 2. Mobile Health (mHealth)

### 2.1. Technologies to Support Dermatology Services

There are various innovative ways to enhance the quality of peripheral dermatology care. These include, but are not limited to, technological methods such as telemedicine, artificial intelligence, and mobile health (mHealth). In teledermatology, telecommunication technologies are used to exchange information regarding skin conditions over distances that a patient would otherwise have to travel [12]. The two main methods are: ‘real-time’ (synchronous) videoconferencing teledermatology, when the patient and dermatologist meet simultaneously in different locations; and ‘store-and-forward’ (asynchronous) teledermatology, in which images are transmitted electronically to a consulting dermatologist [13]. A significant limitation regarding teledermatology is the need for a dermatologist that can be consulted when required. Additional limitations for real-time teledermatology are the need for a stable internet connection and the costs for the necessary infrastructure.

Artificial intelligence can be used to make a computer-based algorithm that is able to make diagnostic decisions regarding skin conditions to a good degree of accuracy [12]. However, these techniques are still in development and only available for a limited number of skin diseases [14].

### 2.2. mHealth to Improve Peripheral Services forSkin Diseases

mHealth refers to the use of mobile communication devices to support health system needs. It can be made available by and for resource managers and health workers, as well as community members, patients, and their caregivers. It can also be used for management purposes, data collection and reporting, decision support, training, emergency referrals, alerts, and supervision [15,16]. In Labrique’s paper on mHealth innovations as health system strengthening tools, the focus is on mHealth applications as point of care decision support for health workers to enhance their capability in diagnosing, treating, and referring people with skin diseases [16]. A systematic review on the feasibility and effective use of mHealth strategies by frontline health workers in developing countries concluded that these tools are potentially an effective means to promote the shifting of essential health services to lower cadres of health workers, but that a balance between the technical requirements and the cost of a system is a vital factor in the scalability of such solutions [17].

There are many mobile phone applications concerning skin diseases. As of August 2017, more than 520 dermatology-related mobile phone applications are available in the Google Play Store and the Apple App Store [18]. The majority of these applications are developed for clinical use such as teledermatology consultation, as self-surveillance and self-diagnosis aids, or as general dermatology references [18,19]. General dermatology reference applications range from an information source for patients to a comprehensive guide for health professionals [19]. Teledermatology is an effective method, as it provides consultations for health professionals in remote and resource-poor areas [20,21]. Some teledermatology applications cover a broad range of diseases, such as ‘Dermassistance’, which combines a mobile phone application with the use of teledermatology (https://www.dermassistance.es/). There is also a variety of disease-specific applications available. In the field of leprosy, for example, there is an initiative aimed at the early recognition of leprosy using image recognition through neural network technology by the Vellore Institute of Technology (http://preleprosy.com/), but this is still in development. 

### 2.3. Development of NLR SkinApp

To increase the capability of peripheral health workers in resource-poor settings to diagnose and treat skin diseases and thus strengthen the health system, the Netherlands Leprosy Relief (NLR) developed an application suitable for Android and Apple devices. To our knowledge, no other similar mobile phone application is available. The aim was not only to support health workers to better serve a large proportion of their patients with common skin diseases, but also to enhance the detection and treatment of less common skin diseases, such as leprosy. Knowing that correct diagnosis and timely treatment help to prevent disabilities, the use of the SkinApp can contribute to reduced suffering caused by dermatological conditions.

The SkinApp uses an algorithm to support the process of diagnosis. It was inspired by the algorithm developed by Mahé for the management of common skin diseases at the primary healthcare level [22,23]. It contains descriptions of skin diseases, supporting photos, as well as treatment and referral advice. The selected skin diseases are a combination of commonly occurring skin diseases, skin diseases that may lead to mortality, HIV/AIDS related skin diseases [24,25], and NTDs manifesting in skin lesions prevalent in sub-Saharan African countries [8,26].

The development of the NLR SkinApp went through three stages: (1) a pilot project using a paper-based algorithm in Nigeria [23]; (2) a pilot of the first version of the mobile phone application in 2015 in Zambezia Province, Mozambique; and (3) an implementation project in Nampula Province, Mozambique, 2017–2018. During all three stages, several dermatology experts with experience in resource-poor areas generously contributed to the content, narrative, and imagery, and provided feedback.

The study in Nigeria was a prospective pilot study to assess the performance of first-line healthcare providers using a paper-based algorithm to support their diagnosis and treatment of seven skin diseases. In this study, 19 patent medicine vendors and 12 traditional healers assessed a total of 4147 patients with skin lesions, and their diagnoses and treatment choices were validated by two independent dermatologists. Overall, the first-line healthcare providers using the algorithm correctly diagnosed and treated or referred 82% of patients presenting with skin lesions. Adding pictorial images of signs and symptoms was believed to further improve the algorithm [23]. On the basis of these findings, it was decided to start the development of a mobile phone application.

In the first version of the SkinApp, which was tested in Zambezia Province, in Mozambique, 21 diseases were included in the algorithm: eight common skin diseases, six NTDs that manifest in skin lesions, and seven HIV/AIDS-related skin diseases (see Table 1). The pilot project was set up to determine the user-friendliness of the application and to identify points for improvement This was done by observing and interviewing SkinApp users through semi-structured interview guides and focus group discussions. The health workers—consisting of one physician, four medical technicians and five nurses—said that they used the application in cases where the diagnosis was clear but the treatment was unknown; whenever they were in doubt; to read through signs and symptoms from diseases in their differential diagnosis; and, when necessary, to go through the algorithm step by step to see if they could come to a diagnosis. They also used the app as a training tool, going through it in their free time. The main findings were that (1) smartphone ownership is very common among health workers in Zambezia; (2) the SkinApp was easy to use after a short introduction about its functionalities; (3) not all skin diseases in the application were relevant in the context of Zambezia Province (e.g., Buruli ulcer); (4) the treatment advice was clear, but not all treatment options were available at the peripheral level in Zambezia Province; (5) a teledermatology function would enhance the usefulness of the application.

Based on the results of the pilot and advancing insight, a second version of the SkinApp was developed. While the first version presented possible diagnoses with a description of signs, symptoms, and treatment advice, the second version aligned much better with the clinical validation of a patient with a skin disease. This was achieved by integrating an improved algorithm that starts with signs and symptoms and their location on a body map, and leads to the most likely diagnosis and treatment advice. In the second version, a number of diseases and supporting photos were added. A feedback button was inserted, which could be developed into a teledermatology function if required. The second version of the SkinApp was implemented in Nampula Province in 2017–2018. A WhatsApp group was formed for SkinApp users to meet the need for a teledermatology option. This enabled them to consult with an experienced Mozambican dermatologist, if needed, though an internet connection is required. Findings from the implementation project confirmed that the SkinApp is more easily accessible than literature and books, and that it is easy to operate. The narrative and illustrative content were considered clear. However, a glossary explaining dermatological terminology would help to improve intelligibility. Adding a reporting option was also mentioned as a possible improvement. These findings helped to further improve the user-friendliness of the SkinApp, but these pilots did not address the performance of the SkinApp as a diagnostic tool.

On the basis of the experiences with the SkinApp in Mozambique and the feedback received from involved dermatologists, a third version has since been developed. Its performance is currently being validated. The third version contains a total number of 29 skin diseases (see Table 1), an improved algorithm, a glossary with frequently-used terminology, and a section on treatment options for frequently-seen skin conditions (broken skin and dry skin).

The second version of the NLR SkinApp is currently available in English and Portuguese for mobile devices with Android as well as iOS operating systems through the Google Play Store (SkinApp) and the Apple App Store (as Skin_App) respectively, free of charge. Once downloaded, the SkinApp can be used offline.

The third version will be released once the current validation study is completed. The validation study is done in clinical settings under appropriate ethical and statistical guidelines. It encompasses concordance testing of the photos used in the application. The accuracy of the SkinApp will be assessed by comparing the diagnosis made by peripheral health workers using the SkinApp, to the diagnosis made by experienced dermatologists. Further evaluation of the use of the third version is planned for in several African countries. The capacity of the health workers to diagnose and treat skin diseases at baseline will be compared to their capacity two years after the introduction of the use of the SkinApp. Furthermore, a comparison will be made between the detection and treatment of skin diseases—specifically neglected tropical diseases—before and after the introduction of the SkinApp.

Once validated, the NLR SkinApp will be made available for use in other countries, where it can be modified to the context by changing the language, adapting the included skin diseases to those prevalent in the area, and using country-specific photos and treatment advices.

## 3. Discussion and Conclusions

Agarwal et al. conducted a systematic review entitled ‘Evidence on feasibility and effective use of mHealth strategies by frontline health workers in developing countries’ [17]. They found that mHealth strategies for peripheral health workers in low-resource countries are potentially an effective means to promote the shifting of essential health services to peripheral levels. What is not known about mHealth strategies is whether they contribute to improved health outcomes, efficiency, or cost-effectiveness. Additional research is needed to determine the added value of mHealth in general, and the SkinApp in particular.

One of the success factors for the adoption of mobile tools identified by Agarwal et al. was the involvement of the health workers throughout the development and implementation process [17]. The SkinApp has been developed in three stages, during which the input of peripheral health workers regarding the use of the SkinApp was obtained. Some of their requests have not yet been integrated into the newest version of the SkinApp, such as the desire for data collection and a teledermatology option. Once the third version of the SkinApp is validated, these functionalities could be added to the application. However, in order to maximize user-friendliness, the aim is to limit the size of the application in megabytes. Furthermore, options to send and receive information not only require an internet connection, but will also need a network of professionals to process the information received and prepare information to be sent. This will increase the cost of the necessary technical support as well as human resources, while a balance between technical requirements and the cost of the system is a vital factor in the scalability of such solutions [17]. The success of an mHealth intervention also depends on the existence of an mHealth platform to facilitate not only the adoption of the tool, but also to guarantee a sustained effective use.

There is a clear need to address the mismatch between the burden of skin diseases at the community level in resource-poor areas and the capability of peripheral health workers to diagnose, treat, and refer people with skin diseases, including skin NTDs. mHealth in general and the NLR SkinApp in particular can help to bridge this gap. The technical requirements and costs to ensure the sustainability of these innovative approaches are likely to increase, especially upon usage in different countries, which requires context-specific adaptation such as the use of the local language and alignment with the epidemiological situation. Therefore, more research is needed to gather evidence on the health outcomes and (cost-) effectiveness of mHealth for skin diseases, including skin NTDs, to ensure a wider use in support of peripheral health workers.

## Figures and Tables

**Table 1:** Skin Diseases in The SkinApp. NTD: neglected tropical disease.

Common Skin Diseases	NTDs Manifesting in Skin Lesions	HIV-Related Skin Diseases	Others
Acne ^1^	Buruli ulcer ^1^	Angular cheilitis ^1^	Albinism
Atopic eczema ^1^	Cutaneous leishmaniasis ^1^	Herpes simplex ^1^	Blistering diseases
Contact eczema ^1^	Leprosy ^1^	Herpes zoster ^1^	Creeping eruption
Impetigo ^1^	Lymphatic filiariasis ^1^	Kaposi sarcoma ^1^	Vitiligo
Pityriasis versicolor ^1^	Mycetoma	Molluscum contagiosum ^1^	
Psoriasis	Onchocerciasis ^1^	Oral candidiasis ^1^	
Seborrhoeic eczema ^1^ (*)	Podoconiosis	Pruritic papular eruption ^1^	
Tinea capitis ^1^	Scabies ^1^		
Tinea corporis ^1^ (*)	Yaws		

^1^ Diseases included since the first version of the SkinApp. (*) also HIV-related.
